# Assessment of Quality of Life and Selected Skin Parameters of People With Pigmentary Lesions: A Pilot Study

**DOI:** 10.1111/jocd.71091

**Published:** 2026-07-23

**Authors:** Anna Bogdanska, Barbara Bogdanska, Mateusz Cybulski, Dariusz Matosiuk

**Affiliations:** ^1^ Doctoral School Medical University of Bialystok Bialystok Poland; ^2^ Department of Integrated Medical Care, Faculty of Health Sciences Medical University of Bialystok Bialystok Poland; ^3^ Doctoral School Medical University of Lublin Lublin Poland; ^4^ Department of Synthesis and Chemical Technology of Pharmaceutical Substances, Faculty of Pharmacy Medical University of Lublin Lublin Poland

**Keywords:** diagnostics, quality of life, skin analysis, skin discoloration, skin parameters

## Abstract

**Background:**

Discoloration is a common skin disorder that significantly affects patients' psychosocial functioning. Understanding the relationship between pigmentary changes and quality of life is essential for comprehensive patient care.

**Aims:**

To evaluate the quality of life and selected skin biophysical parameters in people with pigmentary lesions, with a focus on the relationship between the severity of pigmentary changes, skin parameters, and demographic factors.

**Patients/Methods:**

The study included 30 adults with pigmentary changes. A standardized Dermatology Life Quality Index (DLQI) questionnaire, an author's survey questionnaire, and a numerical satisfaction scale (0–10) were used. The severity of pigmentary changes was assessed using the International Severity Rating Scale, while skin parameters (hydration, oiliness, desquamation, pore size) were measured using the Nati V3 device.

**Results:**

The mean DLQI value was 5.36 ± 5.72 points, indicating a slightly reduced quality of life. The patients showed increased oiliness (*M* = 26.72% ± 22.53%), reduced hydration (Me = 24% in the T‐zone, Me = 20% in the U‐zone), and dilated pores (0.16 mm in 56.7% of subjects). The severity of discoloration (*M* = 19.40 ± 10.19) showed significant differences in T‐zone hydration (*p* = 0.040) and desquamation (*p* = 0.033). Those who rated their health as worse showed significantly reduced quality of life (*p* = 0.011).

**Conclusions:**

Discoloration has a significant impact on quality of life, especially in terms of feelings of attractiveness and mood, and is accompanied by characteristic changes in biophysical parameters of the skin. A holistic approach to treatment should include both dermatological therapy and psychological support.

## 1 Introduction

Discoloration affects over 3 billion people [1, 2, 3]. The connection between discoloration and psychosocial problems is complex, and may be due to an abnormal self‐image, as well as the significant impact of the disease on development in the sphere of socialization. Studies on the psychosocial impact of discoloration have documented dissatisfaction with appearance, embarrassment, shame, and lack of self‐confidence in affected individuals [3]. Social dysfunctions have also been observed, including concerns about sexual contact, avoidance of public appearances, interactions with strangers, and problems with finding work, as well as anxiety, depression, feelings of anger, and lower satisfaction with one's body image [1]. People with discoloration had worse mental health outcomes than those with asthma, epilepsy, diabetes, back pain, arthritis, and coronary artery disease [4].

Quality of life assessment has become a standard measure in most studies. Discoloration can negatively affect a life, so various methods of assessment have been described, including the Dermatology Life Quality Index (DLQI) and a numerical satisfaction scale [5]. The disease is diagnosed on the basis of an interview and examination, which involves a close look at the skin and an assessment of the size of the lesions. Devices are also used that accurately assess the nature and severity of pigmentary changes. These include, for example, a magnifying glass, dermatoscope, Wood's lamp, and tools for assessing skin parameters such as hydration, oiliness, desquamation, pore size, etc. [6, 7, 8].

The aim of the study was to evaluate the quality of life and selected skin parameters of people with discoloration; the following research questions were formulated:

1. What is the level of quality of life of people with discoloration, examined using the DLQI scale, and what is the level of quality of life using the numerical satisfaction scale?
2. What characteristics are indicated by the parameters of skin with discoloration?
3. Is there a relationship between the severity of discoloration and the skin parameters studied?
4. Is there a relationship between quality of life and the skin parameters studied?
5. Is there a relationship between the severity of discoloration and the subjects' quality of life?
6. Do variables such as age, place of residence, self‐assessed health status affect the quality of life of people with discoloration?
7. Do variables such as age, place of residence, and self‐assessed health status affect the skin parameters of people with discoloration?

## 2 Materials and Methods

### 2.1 Participants

Thirty adults with pigmentary changes were included in the study. The majority were women, with one in ten respondents being male. The mean age in the study group was 22.83 years, with a median of 23 years.

### 2.2 Study Design and Procedure

The study was conducted at the Department of Synthesis and Chemical Technology of Medicinal Products of Medical University of Lublin, Poland between 5 May 2025 and 6 June 2025. The study was conducted as an observational cross‐sectional study. Volunteers meeting the inclusion criteria (age ≥ 18 years, with pigmented lesions present) and not subject to the exclusion criteria (current invasive dermatological therapies, lack of consent) were recruited. The study used a diagnostic survey method with the questionnaire technique, relying on the authors' original questionnaire and standardized research tools. The study consisted of two parts carried out during one visit: (1) the questionnaire part, which consisted in filling in the Dermatological Life Quality Index (DLQI) scale and the proprietary survey questionnaire, and (2) the clinical and equipment part, which consisted in assessing the severity of pigmentary lesions with an international scale.

Forty‐five people applied for the study; recruitment was conducted by volunteer selection among people with skin discoloration. The recruitment process was carried out in stages—each reported person was evaluated for compliance with the inclusion criteria and absence of the contraindications specified in the exclusion criteria. As a result of the initial eligibility assessment, 15 people (33.3% of those enrolled) were excluded, which was an important element in ensuring the homogeneity of the study group and the safety of participants. The main reasons for exclusion included: current invasive dermatological therapies, lack of consent to participate in the study, and failure to meet the inclusion criteria. After the selection process, 30 subjects (66.7% of those reported).

Each participant in the study had one visit, during which a comprehensive assessment was implemented, consisting of two parts: a questionnaire part and a clinical and equipment part.

In the questionnaire portion, participants filled in surveys on the spot, in the presence of a researcher, thus ensuring a 100% return rate and eliminating data loss. Each subject was evaluated one at a time to avoid variability related to time of day, sunlight exposure, or other factors affecting color perception. The use of standardized procedures allowed us to obtain reliable and comparable results of discoloration severity.

### 2.3 Research Tools

The pilot study presented here used a comprehensive set of standardized and validated measurement tools, including both standard psychometric scales and state‐of‐the‐art diagnostic devices.

The Dermatology Life Quality Index (DLQI) is a tool consisting of 10 single‐choice questions, each of which addresses specific aspects of the patient's functioning over the past 7 days. The questionnaire covers 6 major areas of the patient's life that may be affected by skin diseases: symptoms and feelings (questions 1 and 2), daily activities (questions 3 and 4), leisure and sports (questions 5 and 6), work or school (question 7), personal and intimate relationships (questions 8 and 9), and the impact of the treatment used (question 10). Each question is scored on a four‐point scale (from 0 to 3 points), where 0 means “not at all” or “not applicable”, 1 “a little”, 2 “quite a lot”, and 3 “a lot”. Total DLQI scores range from 0 to 30 points, where higher scores indicate a greater impact of the disease on quality of life. Interpretation of the results is based on a predetermined scale: 0–1 point indicates normal quality of life (no impact), 2–5 points indicate low impact on quality of life, 6–10 points indicate medium impact, 11–20 points indicate high impact, and 21–30 points indicate very high impact on patient functioning [9, 10, 11].

The authors' survey questionnaire is a tool developed specifically for this study to collect detailed demographic and clinical data from participants. This questionnaire is not a standardized and internationally validated tool, but a diagnostic tool tailored to the specific research objectives of the pilot study.

The questionnaire includes two main categories of questions. The first category deals with demographic and socio‐demographic data and includes questions on gender, age, place of residence, housing conditions, and education level. The second category of questions refers to clinical data associated with discoloration, including questions about the duration of pigmentary lesions, the form of discoloration, subjective assessment of lesion severity, location of discoloration on the body, previous treatment attempts, and self‐assessment of the condition.

The five‐item numerical scale of life satisfaction (0–10) is a clinical tool particularly useful in the pilot study due to its simplicity, ease of interpretation and the ability to measure changes over a short period of time [10].

The scale included an assessment of the impact of pigmentary changes on 5 isolated aspects of functioning: (1) ability to perform at work, (2) daily activities, (3) mood, (4) social life, and (5) sense of being attractive. Each aspect was rated on a scale of 0 to 10 points, where 0 meant no impact or extremely positive feelings, and 10 meant the maximum negative impact of skin lesions on a particular aspect of functioning [10].

The International Melasma Severity Scale (IMSS) is a standardized tool for objectively assessing the severity and extent of pigmented lesions on the skin. The most commonly used scale in clinical and research practice is the Melasma Area and Severity Index (MASI) and its modifications, such as the modified Melasma Area and Severity Index (mMASI) or Melasma Severity Index (MSI) [12]. Cronbach's alpha values were within the range indicating good or excellent internal consistency (Cronbach's alpha values above 0.80).

The Nati V3 device is an advanced skin analysis system featuring a high‐resolution camera (HD Ready, ×40 magnification) and advanced optical‐physical technology with multi‐source illumination for precise measurement of eight biophysical skin parameters.

Nati V3 can measure the following biophysical parameters of the skin:

1. Oiliness (sebum): Measured using a specialized SEBUM STRIP that is adhered to the skin of the forehead and nasal area for 5 s.
2. Hydration: The water content of the stratum corneum is measured in two regions of the face—the T‐zone (forehead, nose, chin—more lipid‐active areas) and the U‐zone (cheeks—areas more prone to dryness).
3. Desquamation/corneum strip: The measurement is performed using a specialized CORNEUM STRIP adhered to the cheek, which absorbs dead skin cells.
4. Pore size: Measurement of pore diameter and the degree of pore clogging (the content of sebum and dead cells in the pores) is carried out in the nose and forehead area using HD optical image analysis.
5. Other parameters: The device is also capable of measuring skin texture, elasticity, vascular changes (redness, vessels), pigmentation changes, and wrinkle depth, although this study focused mainly on the parameters of oiliness, hydration, desquamation, and pore size [13].

### 2.4 Statistical Analysis

The statistical analysis was performed using Statistica 13.3 PL software from StatSoft Polska Sp. z o.o. (Kraków, Poland). Normality of distribution was checked with the Shapiro–Wilk test. The results were presented using descriptive statistics, such as arithmetic mean, standard deviation, median, and minimum and maximum values. The Mann–Whitney *U* test was used for two‐group comparisons, while the Kruskal–Wallis test with post hoc analysis was used for multigroup comparisons. A significance level of *p* < 0.05 was adopted.

## 3 Results and Discussion

### 3.1 Socio‐Demographic Characteristics of the Participants

30 participants were involved in the study (27 women—90%, 3 men—10%). The age of respondents ranged from 18 to 41 (*M* = 22.83 ± 3.91; Me = 23 years). The majority of respondents lived in cities (62%; *n* = 23), while 38% (*n* = 7) lived in rural areas. The vast majority of respondents (53%) rated their housing conditions as good. Taking into account the level of education, the largest number of respondents had a university degree (*n* = 17.56), slightly fewer had secondary education (*n* = 11.36), and 1 person had primary and vocational education each (3%). Detailed data is shown in Table 1.

**TABLE 1:** Socio‐demographic characteristics of respondents.

Variable	Category	*n*	%
Sex	Women	27	90
	Men	3	10
Age (years)	Scope	18–41	—
	Mean value ± SD	22.83 ± 3.91	—
	Median	23	—
Place of residence	City	23	62
	Village	7	38
Housing conditions	Very good	16	53
	Good	11	37
	Average	3	10
Level of education	Higher education	17	57
	Secondary education	11	37
	Primary education	1	3
	Occupational	1	3

### 3.2 Quality of Life Assessment

Quality of life measured using DLQI showed a mean value of 5.36 ± 5.72 and a median of 4.63, corresponding to a slightly reduced quality of life. Most people (53%) had a score indicating a slight reduction in quality of life (2–5 points on the DLQI scale) (Table 2). Discoloration had the least impact on work performance and daily activities (*M* = 7.97 points on the numerical satisfaction scale). The most strongly felt limitations were in the feeling of being attractive (*M* = 4.17) and mood (*M* = 4.3).

**TABLE 2:** Quality of life ranges.

Quality of life ranges	*n*	%
Normal quality of life	3	10
Slightly reduced quality of life	16	53
Moderately reduced quality of life	8	27
Severely reduced quality of life	2	7
Significantly reduced quality of life	1	3
Total	30	100

Statistical analysis revealed significant differences in quality of life according to self‐assessment of health (*p* = 0.011), where those who rated their health as worse showed significantly reduced quality of life indicators.

Analysis of quality of life by gender showed that women scored *M* = 5.30 ± 4.87 points, while men scored *M* = 6.00 ± 1.73 points. A greater variation in results was observed among women (Table 3).

**TABLE 3:** Descriptive statistics of quality of life of men and women.

Quality of life according to DLQI	*n*	Min.	Max.	*M* ± SD	Me
Woman	27	0	24	5.30 ± 4.87	4
Man	3	5	8	6.00 ± 1.73	5

Abbreviations: *M*, mean; Max., maximum; Me, median; Min., minimum; SD, standard deviation.

### 3.3 Numerical Scale of Life Satisfaction

The assessment of the impact of skin lesions on various aspects of psychosocial functioning showed mixed results. The greatest impact of skin lesions was noted on the ability to work (*M* = 7.97 ± 3.12; Me = 9.5), while slightly lower on daily activities (*M* = 7.20 ± 3.12; Me = 8). Respondents' social life was also limited by skin lesions (*M* = 6.27 ± 3.10; Me = 7). The least direct effect of pigmentation changes was observed on mood (*M* = 4.30 ± 2.85; Me = 4) and a sense of being attractive (*M* = 4.17 ± 2.91; Me = 4), although both parameters indicated a significant psychological impact of the disease on the subjects (Table 4).

**TABLE 4:** Descriptive statistics of the numerical scale of life satisfaction of subjects with discoloration.

Numerical scale 0–10	*n*	*M*	SD	Min.	Max.	Me
Skin lesions limited my daily activities	30	7.20	3.12	0	10	8
Skin lesions made me unable to do my job	30	7.97	3.12	0	10	9.5
Skin lesions adversely affected my mood	30	4.30	2.85	0	10	4
I have been limiting my social life due to skin lesions	30	6.27	3.10	0	10	7
I feel less attractive due to skin lesions	30	4.17	2.91	0	10	4

Abbreviations: *M*, mean; Max., maximum; Me, median; Min., minimum; SD, standard deviation.

### 3.4 Severity of Pigmentary Changes

The mean score for the severity of pigmentary lesions was 19.40 ± 10.19 points (Me = 21), indicating a moderate severity of the disease in the analyzed population. The detailed distribution of severity based on the international rating scale is shown in Table 5.

**TABLE 5:** The severity of pigmentary changes in the studied population.

Severity of pigmentary changes	*n*	%
1–18 points (mild)	12	40
19–30 points (moderate)	13	43
31–38 points (severe)	5	17
> 39 points (very severe)	0	0
Total	30	100

### 3.5 Skin Parameters

Skin oiliness measurements showed a median of 19.3% (range: 3.3%–77.1%). The results indicated increased oiliness, characteristic of hyperpigmented skin, as a consequence of excessive sebaceous gland activity. Moisture levels tested in two zones showed in the T‐zone (forehead): Me = 24% (below normal), while in the U‐zone (cheek): Me = 20% (low hydration). The results suggest that despite the increased oiliness, the skin of the subjects showed reduced hydration, which is typical of this type of lesion. The average pore diameter was 0.16 mm, which classifies them as dilated. Pore dilation is related to the accumulation of sebum and dead skin cells at the mouths of hair and sebaceous follicles. The level of desquamation demonstrated a median of 7.74%, which is within the norm for well cared for skin. This result indicates a normal desquamation process in the studied population (Table 6).

**TABLE 6:** Evaluation of skin parameters of persons with discoloration.

Skin parameters	Variable	*n*	%	*M* ± SD (Min–Max)
Oiliness level	Dry skin (0%–10.99%)	9	30	26.72 ± 22.53 (3.3–77.1)
	Skin prone to dryness (11%–14.99%)	3	10	
	Proper oiling of the skin (15%–20.99%)	4	13	
	Skin prone to oiliness (21%–25.99%)	6	20	
	Oily skin (26%–100%)	8	27	
Desquamation	Good (0%–11.99%)	28	93	8.45 ± 2.58 (3.7–17.7)
	Normal (12%–20.99%)	2	7	
	Excessive (21%–100%)	0	0	
Pores	Small pores (0–0.1 mm)	1	3	0.16 ± 0.03 (0.08–0.25)
	Normal (0.11–0.15 mm)	12	40	
	Dilated above 0.16 mm	17	57	
T‐zone hydration	Low (0%–24%)	17	57	25.80 ± 14.44 (10.0–63.0)
	Normal (25%–40%)	8	27	
	Good (41%–65%)	5	17	
U‐zone hydration	Low (0%–24%)	18	60	25.60 ± 13.23 (10.0–60.0)
	Normal (25%–40%)	8	27	
	Good (41%–65%)	4	13	

Abbreviations: *M*, mean; Max., maximum; Min., minimum; SD, standard deviation.

### 3.6 Relationship Between Skin Parameters and Severity of Discoloration

The statistical analysis using the Kruskal‐Wallis test showed no statistically significant differences between skin parameters and the severity of discoloration. In contrast, statistical significance was shown for T‐zone desquamation and hydration (Table 7). The lack of significant correlations suggests that the severity of pigmentary changes may be independent of measurable skin parameters.

**TABLE 7:** Skin parameters depending on the severity of discoloration.

Skin parameters	Severity of pigmentary changes									Kruskal‐Wallis test	
	Mild			Moderate			Severe			*H*	*p*
	Me	Min.	Max.	Me	Min.	Max.	Me	Min.	Max.		
Oiliness	19.3	3.3	73.9	21.45	5.4	77.1	16.5	12.8	23.0	0.132	0.936
Desquamation	9.09	7.2	12.5	8.90	3.7	17.7	7.09	3.92	7.64	6.793	0.033*
Pores	0.15	0.13	0.19	0.17	0.08	0.25	0.15	0.14	0.25	1.651	0.438
Hydration T‐zone	32.0	10.0	51.0	24.0	10.0	63.0	12.0	10.0	16.0	6.437	0.040*
Hydration U‐zone	28.0	10.0	48.0	24.0	12.0	60.0	16.0	16.0	38.0	0.753	0.069

Abbreviations: *H*, Kruskal–Wallis test statistic; Max., maximum; Me, median; Min., minimum; *p*, *p*‐value.

* Statistically significant value.

### 3.7 Impact of Self‐Assessed Health Status on Life Quality

Comparison using the Mann–Whitney *U*‐test showed statistically significant differences in the perceived impact of skin lesions between those who rated their health as good or poor in terms of limitations in daily activities (*p* = 0.015), limitations at work (*p* = 0.010), and limitations in social life (*p* = 0.009) (Table 8). Those who rated their health positively showed higher median values, indicating a greater perception of the limitations of discoloration. This may suggest that people with well‐assessed health are more aware of the direct impact of skin lesions on their performance.

**TABLE 8:** Satisfaction with life as measured by a numerical scale in relation to self‐assessed health of people with discoloration.

Skin parameters	Health status self‐assessment						Mann–Whitney *U*‐test	
	Good, very good			Bad, very bad			Z	*p*
	Me	Min.	Max.	Me	Min.	Max.		
The skin lesions limited my daily activity	9	0	10	5	2	5	2.421	0.015*
The skin lesions caused me to be unable to do my job	10	0	10	4	0	9	2.588	0.010*
My skin lesions adversely affected my mood	4	0	10	1	0	8	1.809	0.071
Due to my skin lesions I was limiting my social life	7	1	10	4	0	5	2.615	0.009*
I feel less attractive due to skin lesions	4	0	10	1	0	8	1.503	0.133

Abbreviations: Max., maximum; Me, median; Min., minimum; *p*, *p*‐value; *Z*, Mann–Whitney *U*‐test statistic.

* Statistically significant value.

### 3.8 Skin Parameters Based on Health Self‐Assessment

Analysis with the Mann–Whitney *U* test showed no statistically significant differences in skin parameters (oiliness, desquamation, pores, hydration in both zones) between subjects with good and poor self‐assessed health (all *p* > 0.05).

### 3.9 Quality of Life for People With Skin Discoloration

The mean DLQI value was 5.93 points, indicating a slightly reduced quality of life in the studied population. More than half of the respondents (53%) obtained DLQI values indicating a slight reduction in quality of life. (2–5 points) [14, 15].

Women accounted for 90% of the participants, which is consistent with the epidemiology of pigmentary disorders. Differences in DLQI scores between women (*M* = 5.30 ± 4.87) and men (*M* = 6.00 ± 1.73) did not demonstrate statistical significance, but greater variation was observed among women, suggesting greater heterogeneity of psychosocial experiences in women [3].

The greatest impact of discoloration was observed on the ability to work (*M* = 7.97 ± 3.12), while the least impact was observed on the feeling of being attractive (*M* = 4.17 ± 2.91) and mood (*M* = 4.30 ± 2.85). This pattern is consistent with studies showing that pigmentation disorders lead to negative self‐perception, shame, and lack of self‐confidence, which can manifest particularly in the emotional and esthetic areas [3].

Our results (DLQI = 5.93) are significantly lower than in Pakistani studies (17.08 ± 5.22) [16]. Values similar to ours were observed in the study by Ghale et al., where the mean DLQI was 6.16 points, which may suggest that geographic and cultural differences in the perception of discoloration may affect patients' well‐being [7].

Studies conducted in different geographic populations reveal significant disparities in the perception and impact of pigmentary disorders on patients' quality of life. A Pakistani study by Raafel et al. was classified as having a “very large effect” on quality of life, particularly in women and patients with a severe form of the disease [17]. In contrast, the European and Polish populations show a milder impact of discoloration (DLQI = 5.93), which may be explained by the younger age of the patients (mean value of 22.83 years), lower severity of pigmentary changes, and other cultural and esthetic norms related to skin color [3, 16].

The Greek study, conducted by Platsidaki et al. showed that vitiligo and post‐inflammatory hyperpigmentations showed higher DLQIs (mean DLQI for vitiligo of ca. 12.5) compared to melasma [3].

The Vitiligo Impact Patient (VIPs) scale was 27.3 (0–60 scale), with the highest scores observed in India (40.2) and Brazil (29.7). This indicates that in addition to the biological features of the disease, social and cultural factors, such as social stigma, have a significant impact on the psychosocial burden of pigmentary disorders [18].

### 3.10 Psychosocial Impact of Discoloration

Analysis of the impact of self‐assessed health status on quality of life showed statistically significant differences (*p* = 0.011). Those who rated their health as worse showed reduced DLQI scores, which is paradoxical from the perspective of skin lesions. Scores on the numerical satisfaction scale showed that those with a positive self‐assessment of health suggest a higher awareness and greater impact of discoloration on daily functioning.

The scientific literature confirms the significant psychosocial impact of discoloration on patients. Studies have shown that the incidence of depression and anxiety among melasma patients is 33% and 22%, respectively, while the rates are much lower in the general population. In addition, patients with discoloration show higher levels of anxiety compared to healthy individuals (7.47 ± 4.53 vs. 6.06 ± 3.59; *p* = 0.006) [3, 19].

The study by Dabas et al. showed that the frequency of anxiety and depression in patients with melasma (11.6% and 12.8%) is significantly lower than in patients with vitiligo (21% and 27%) or acquired dermal macular hyperpigmentation (ADMH) (18.7% and 24.1%), suggesting that the degree of visibility of the lesions and the ability to mask pigmentary changes may affect the psychological state of patients [4]. Our observations of a significant difference in quality of life according to self‐reported health status (*p* = 0.011) are consistent with studies that suggest that patients who are more aware of their health disorders may perceive discoloration as a greater threat to their overall psychophysical state.

A Greek study by Platsidaki found that patients with melasma showed significantly higher levels of anxiety (7.47 ± 4.53) compared to healthy subjects (6.06 ± 3.59; *p* = 0.006), although depression was not significantly different between groups [3]. A Chinese meta‐analysis by Chen et al. revealed that depression occurs in 33.3% (95% CI: 27610–39 057) of patients with melasma, and anxiety in 21.6% (95% CI: 16.595–26.587) [19].

Interestingly, age ≥ 45 years was a protective factor for depression in patients with melasma, while high BMI and poor quality of life were independent risk factors for depression [19].

Deshpande's research found that 54% of melasma patients reported that the pigmentation disorder was the result of or exacerbated by stress. This bidirectional relationship between stress and melasma is mediated by the hypothalamic–pituitary–adrenal (HPA) axis and the local cutaneous CRF‐POMC‐corticosteroid axis, while melanocytes are reactive to stress hormones [20].

### 3.11 Biophysical Parameters of the Skin

The results of measuring skin parameters revealed a characteristic biophysical profile of skin with discoloration. The average oiliness was 26.72% ± 22.53%, indicating significant variability in the population, with 26.7% of subjects having oily skin (> 26%). This oiliness pattern is consistent with knowledge of the pathophysiology of melanogenesis, where sebocytes play an important role in melanocyte activation and melanin production. Studies have shown that UV‐irradiated sebocytes produce and activate factors such as α‐MSH (hormonal melanocyte‐stimulating hormone), EDN1 (endothelin‐1), b‐FGF (basic fibroblast growth factor), and SCF (stem cell factor), which directly stimulate melanogenesis. In addition, sebocytes contribute to a local pro‐inflammatory and photo‐aging environment, which promotes the maintenance of discoloration [21].

The paradox observed in our own population study—the presence of increased oiliness combined with reduced hydration in the T‐zone (Me = 24%) and U‐zone (Me = 20%) is noted. The study by Voegeli et al. showed that skin pigmentation does not correlate with overall skin barrier parameters as measured by TEWL (Transepidermal Water Loss), but is strongly associated with stratum corneum hydration. This suggests a specific skin barrier dysfunction in pigmentary disorders, which may be related to impaired lipidization or filaggrin (FLG) dysfunction [22].

Given that the majority of our patients live in cities (62%), there is the possibility of less UV exposure compared to populations from tropical or Mediterranean areas, as studied by Basucal [23].

Skin hydration parameters showed reduced values, especially in the T‐zone (Me = 24) and U‐zone (Me = 20%). The pattern where reduced hydration is observed despite increased oiliness is typical of skin with pigmentation disorders and may be associated with skin barrier disorders [24].

The pore diameter was 0.16 mm (Me = 0.16 mm), which classifies the pores as dilated (> 0.16 mm) in 56.7% of the subjects. The enlargement of pores is a consequence of the accumulation of sebum and dead skin cells at the mouths of hair and sebaceous follicles. The relationship between pore size and melanogenesis may be explained by the fact that sebocytes are most abundant in the central regions of the face (forehead, nose), which are also predisposed to pore enlargement and are less prone to post‐inflammatory discoloration [21].

The level of desquamation showed a median of 7.74%, which is within the norm for well cared for skin (6%–10%). This result is surprising, as it indicates that despite the pigmentation changes, the natural desquamation process remains normal. This may suggest that pigmentary abnormalities are not directly related to keratinization disorders or desquamation of the stratum corneum [24].

The characteristic profile of biophysical parameters in our population differs from the norms established in population studies. While the average oiliness in patients with discoloration (26.72% ± 22.53%) is similar to the values observed in the general population, reduced hydration values in the T‐zone (Me = 24%) and U‐zone (Me = 20%) indicate skin barrier dysfunction. These observations are consistent with the study by Flori et al., which showed that skin with melasma is characterized by impaired integrity of the stratum corneum and delayed rates of barrier regeneration [21]. Dabas et al. highlighted the importance of anatomical location for biophysical parameters—the forehead (T‐zone) naturally exhibits higher oiliness and lower hydration, which may explain the changes observed in our population [4].

### 3.12 Relationship Between Severity of Discoloration and Skin Parameters

Statistical analysis using the Kruskal–Wallis test showed significant differences between T‐zone desquamation (*p* = 0.033) and T‐zone hydration (*p* = 0.040) and the severity of discoloration. Studies on a broader population indicate that skin pigmentation has no effect on overall skin barrier properties (such as Transepidermal Water Loss (TEWL)), but is associated with hydration [22]. Our results suggest a more complex relationship, where the severity of discoloration may be related to subtle changes in hydration and desquamation, but not to overall oiliness or pore size parameters.

The lack of a strong association between skin parameters and the severity of discoloration in our study may suggest that biophysical properties of the skin are not the main determinants of the severity of pigmentary disorders. Instead, factors such as UV exposure, hormonal factors (especially in women), genetics, and inflammatory parameters may play a more important role in the pathophysiology of discoloration.

Our observations of increased oiliness in patients with discoloration (26.7% of subjects had oily skin) can be explained by the study by Flori et al., which showed that sebocytes after UVA irradiation produce and activate pro‐melanogenic factors such as α‐MSH, EDN1, b‐FGF and SCF [21]. Noteworthy are the recent results by Lee et al. which suggest that sebum itself may have melanogenesis‐inhibiting properties by reducing tyrokinase activity [25].

### 3.13 Effects of Age, Location and Self‐Assessed Health Status

The study population was relatively young (age: 18–41; *M* = 22.83 ± 3.91), which deviates from the typical profile of patients with melasma or other pigmentary disorders. This relatively young population may explain the moderate severity of pigmentary changes (*M* = 19.40 ± 10.19 points) and moderate impact on quality of life. Literature evidence suggests that the severity of discoloration changes with age, with patients with lesions lasting more than 2 years showing higher levels of depression. For this reason, longitudinal studies involving older populations may reveal more significant psychosocial consequences [3].

It was also observed that 62% of the respondents lived in cities, while 38% lived in rural areas. Differences in access to dermatological care can affect treatment outcomes and patient satisfaction. However, in our study, we did not conduct an analysis of the direct effect of residence location on skin parameters or the severity of discoloration [26].

The high proportion of women in our population (90%) is consistent with the epidemiology of discoloration, although the study by Baskaran et al. in the Indian population showed a slightly lower female dominance (67.5%), which may suggest that in European populations, pigmentary disorders may be more related to the psychosocial and esthetic aspects in women [23]. The average age of our patients (22.83 years) is much lower than in typical melasma studies (average 34.6 years in the study by Baskaran et al.), which may affect the interpretation of our results and suggests that additional factors favoring earlier manifestation of discoloration may be at work in our population. The study by Zhao et al. showed that pregnancy history and contraceptive use are important risk factors, which is worth considering in future prospective studies on the Polish population [27].

Also noteworthy is the discovery that thyroid disorders (dysthyroidism) were demonstrated in 21% of melasma patients, and vitamin D deficiency in 22% of patients. These observations suggest a link between broader endocrine dysfunction and discoloration and may have implications for future therapeutic strategies [3].

### 3.14 Limitations of Own Studies

This study has several important limitations that must be considered when interpreting the results. First, the small sample size (*n* = 30) limits the possibility to generalize the results to the entire population of people with discoloration. Second, the study group was dominated by women (90%), making it difficult to infer men's experiences with discoloration. Third, the study was cross‐sectional, making it impossible to establish causal relationships among variables. Fourth, the Nati V3 device used for measuring skin parameters, while reliable, has not been validated for all skin populations, especially for skin with discoloration.

In addition, the study did not take into account important factors that could affect the results, such as: UV exposure frequency; hormonal status and use of hormonal contraceptives; anti‐pigmentation therapies used; genesis of discoloration; ethnic background and genetic predisposition; other health conditions that may affect psychosocial functioning.

### 3.15 Suggested Directions for Further Research

The results of this study have several important clinical implications. First, they clearly demonstrate that pigmentary disorders have a significant impact on psychosocial functioning, especially in terms of feelings of being attractive and mood. For this reason, a holistic approach to patients with discoloration should include not only dermatological treatment but also psychological evaluation and support.

Second, the characteristic profile of skin parameters (increased oiliness, reduced hydration, enlarged pores) in hyperpigmented individuals suggests that sebocytes and hair‐sebum unit dysfunction may be involved in the pathogenesis of pigmentary disorders. These observations may have implications for the development of new therapies aimed at modulating sebocyte activity and restoring skin barrier homeostasis.

Third, the lack of a significant association between most skin parameters and the severity of discoloration suggests that biophysical skin properties may not be the determinants that originally determined the severity of pigmentary changes. Instead, hormonal and immunological factors and UV exposure may play a more important role.

In conclusion, discolorations have a significant impact on the quality of life of adults, especially in terms of psychosocial aspects, and these lesions are accompanied by characteristic biophysical parameters of the skin, including increased oiliness, reduced hydration, and dilated pores. The study provides a starting point for further, more comprehensive research into the pathophysiology and psychosocial impact of discoloration on patients' health.

## Author Contributions

Conceptualization, B.B. and D.M.; Data curation, A.B. and B.B.; Formal analysis, A.B. and B.B.; Funding acquisition, B.B. and D.M.; Investigation, A.B. and B.B.; Methodology, B.B. and D.M.; Project administration, B.B.; Writing – original draft, A.B. and B.B.; Writing – review and editing, M.C. and D.M.; and Supervision, M.C. All authors have read and agreed to the published version of the manuscript.

## Funding

The APC was funded by the Medical University of Bialystok.

## Ethics Statement

The authors confirm that the ethical policies of the journal, as noted on the journal's author guidelines page, have been adhered to and the appropriate ethical review committee approval has been received. The study was approved by the Bioethics Committee of the Medical University of Lublin (resolution No. KB‐0024/24/02/2026, approval date: 12 February 2026). Informed consent was obtained from all subjects involved in the study.

## Conflicts of Interest

The authors declare no conflicts of interest.

## Data Availability

The raw data supporting the conclusions of this article will be made available by the authors on request.
